# Efficacy and safety of ketamine and esketamine for the prevention of postanesthetic shivering in cesarean delivery: a systematic review and meta-analysis

**DOI:** 10.3389/fmed.2026.1845684

**Published:** 2026-06-26

**Authors:** Ke Yang, Qin Liu, Liqiong Zhang, Lei Wang, Feng Xiong, Jie Song, Qun Zhou, Yongbao Peng

**Affiliations:** 1 Department of Anesthesiology, Zigong Maternal and Children Health Care Hospital, Zigong, China; 2 Department of Anesthesiology, Jiangxi Maternal and Child Health Hospital, Nanchang, China; 3 Graduate School of Nanchang University, Nanchang, China

**Keywords:** cesarean delivery, esketamine, ketamine, meta-analysis, shivering

## Abstract

**Purpose:**

Shivering is a common and undesirable complication associated with cesarean delivery under intrathecal anesthesia, which causes discomfort in patients and may interfere with vital signs monitoring. The main purpose of this meta-analysis was to evaluate the efficacy and safety of ketamine and esketamine for the prevention of postanesthetic shivering.

**Methods:**

We performed a systematic search of PubMed, Embase, Web of Science, CENTRAL, CNKI, and Wan Fang databases for randomized controlled trials (RCTs) evaluating the efficacy of ketamine and esketamine in preventing shivering in women undergoing cesarean delivery. The primary outcomes of this analysis were the incidence and the severity of postanesthetic shivering. Secondary outcomes were the incidence of adverse events. We integrated traditional meta-analysis with logistic regression to establish the dose-response relationships.

**Results:**

A total of 12 RCTs involving 1683 patients were included; most are assessed as low risk or with some concerns, and the quality of the evidence is mostly low and moderate. The incidence of postanesthetic shivering during cesarean delivery was significantly lower in the ketamine and esketamine (RR: 0.29; 95% CI: 0.20–0.42; *P* < 0.00001) groups than in the placebo group, particularly for grade 2–4 shivering. Multivariable meta-regression and logistic regression showed that the preventive efficacy of ketamine and esketamine against shivering was significantly correlated with dose. Ketamine and esketamine have an advantage in reducing the incidence of nausea and vomiting (RR: 0.52; 95% CI: 0.38–0.71; *P* < 0.0001), hypotension (RR: 0.36; 95% CI: 0.20–0.66; *P* = 0.001), and bradycardia (RR: 0.47; 95% CI: 0.30–0.73; *P* = 0.0009) compared with a placebo. However, the ketamine and esketamine groups exhibited significantly higher incidences of hallucination (RR: 11.19; 95% CI: 3.46–36.19; *P* < 0.0001) and nystagmus (RR: 10.58; 95% CI: 3.76–29.75; *P* < 0.00001) compared with the placebo group, and these adverse events are dose-dependent.

**Conclusion:**

Prophylactic application of ketamine and esketamine could be efficacious for reducing postanesthetic shivering during cesarean delivery. However, the dose-dependent increases in hallucination and nystagmus highlight the need to balance preventive efficacy against psychiatric adverse events when selecting the optimal dose. Additional convincing evidence is required to prove their value and safety in preventing shivering for obstetric patients.

**Systematic review registration:**

https://www.crd.york.ac.uk/prospero/, identifier CRD420251076816.

## Introduction

Shivering is a common complication of cesarean delivery under intrathecal anesthesia, which is often described as an unpleasant and anxious experience, and affects approximately half of parturients undergoing cesarean delivery (1, 2). Shivering can interfere with vital signs monitoring, and the physical movements associated with shivering can disrupt the interpretation and judgment of heart rate, blood pressure, and electrocardiogram (3, 4). More importantly, vigorous shivering can increase the metabolism of parturients, resulting in a significant increase in maternal cardiopulmonary load and oxygen consumption and potentially leading to hypoxemia and lactic acidosis, even threatening maternal safety (5, 6).

Intrathecal anesthesia preferentially blocks sympathetic nerves, thereby inducing vasodilation that redistributes core heat to peripheral tissues and rapidly lowers core temperature. Furthermore, it disrupts central thermoregulation by blocking afferent temperature signals to the hypothalamus, which in turn lowers the shivering threshold. As sensory afferents are blocked, the thermoregulatory center compensates solely through skeletal muscle contractions in unblocked regions, producing the shivering response (2, 7–9). Previous studies have reported that non-pharmacological measures were used to avoid postanesthetic shivering, such as increasing operating room temperature, warming the intravenous fluid, and using heated blankets (10, 11). Pharmacologic interventions remain the most popular methods for the prevention and treatment of shivering (8). Opioids (e.g., fentanyl, morphine, meperidine, or tramadol) and α_2_-adrenergic agonists (e.g., dexmedetomidine) were demonstrated to have anti-shivering effects, while adverse events like excessive sedation, respiratory depression, nausea, and vomiting were found in the clinical application (2, 5, 12). Ketamine is a competitive *N*-methyl-*d*-aspartate (NMDA) receptor antagonist that is commonly applied as an anesthetic drug in cesarean delivery. Esketamine is a more effective S-enantiomer of ketamine, and its anesthetic and analgesic effects are three to four times higher than those of (R)-ketamine and have fewer adverse events than ketamine (13, 14).

Recent studies have demonstrated that ketamine exerts a preventive effect against postanesthetic shivering across various surgical procedures, and its efficacy is equivalent to that of tramadol; however, there are discrepancies regarding whether ketamine can prevent shivering after intrathecal anesthesia in cesarean delivery (15–17). Given the inconsistent findings and the lack of a meta-analysis addressing esketamine for shivering prevention, it is worthwhile to conduct a meta-analysis of the impact of esketamine and ketamine on the efficacy and safety of postanesthetic shivering during cesarean delivery.

## Methods

This analysis was designed in accordance with the Preferred Reporting Items for Systematic Reviews and Meta-Analysis (PRISMA) standards and has been registered with the International Prospective Register of Systematic Reviews (PROSPERO) website (CRD420251076816).

### Search strategy for literature

A systematic search was conducted across six databases (PubMed, Web of Science, Embase, Cochrane Library, CNKI, and Wan Fang) for the relevant articles published prior to 9 May 2026. We used the Medical Subject Headings (MeSH) with the free words like “ketamine,” “esketamine,” “shivering,” and “cesarean delivery” to search for studies, and the search formula was shown in Supplementary Table 1. At the same time, we conducted an additional review of the references from the selected papers. The study design was restricted to randomized controlled trials. Our research did not need approval from the ethical committee.

### Study selection and eligibility criteria

The procedure of study selection commenced with the elimination of duplicated records. Subsequently, two authors (Y.K. and L.Q.) checked the titles and abstracts of the remaining publications separately. Following the inclusion criteria, the same authors conducted a full-text review to select appropriate research, with any discrepancies decided by the third author. The inclusion criteria were as follows: (1) study type: randomized controlled trials; (2) participants: adult patients undergoing cesarean delivery; (3) intervention: prophylactic use of ketamine and esketamine compared with saline. The exclusion criteria were delineated as follows: (1) reviews, letters, meta-analyses, comments, retrospective studies, case reports, animal experiments, duplicate reports, unpublished and ongoing studies; (2) insufficient research data or unavailable full text; (3) any language aside from English or Chinese.

### Data extraction

The following data were gathered from each included study: (1) general knowledge: initial author, study year and period, country, and sample sizes of the RCTs; (2) demographic information: age, gestational age, body mass index (BMI), and diagnostic criteria for shivering; (3) intervention: details of the administration of ketamine and esketamine, anesthesia selection and medication; (4) primary outcome: the incidence and severity of postanesthetic shivering; (5) secondary outcome: adverse events (e.g., nausea and vomiting, hypotension, bradycardia, hallucination, and nystagmus).

### Quality assessment

Two authors (Y.K. and Z.L.Q.) independently used the revised Cochrane Collaboration’s risk of bias assessment tool (RoB2.0) to assess the quality of included RCTs, which better identifies and analyzes potential risk factors. The systematic tool assesses the risk of bias in RCTs within the five items, and each item includes several signaling questions with general responses ranging from “yes,” “probably yes,” “no,” “probably no,” and “no information.” The measured risk of bias in signaling questions in each item is estimated as “low risk,” “some concerns,” or “high risk.” Consequently, the overall bias of each study is established by considering the cumulative biases. The GRADE-pro approach assessed the certainty of evidence for the primary outcomes of the included RCTs. Any disagreements were resolved during the discussion or consultation with the third author if needed.

### Statistical analysis

The Review Manager 5.4.1 version was employed in this analysis. The effect size (ES) for dichotomous variables was defined using the risk ratio (RR) and 95% confidence interval (95% CI). On the other hand, standardized mean difference (SMD) and 95% CI were chosen as the ES for continuous variables. The significance value was established at 0.05 in this meta-analysis. To assess the presence and degree of inter-study heterogeneity, the heterogeneity index (I^2^) value and Q test were utilized. Under conditions of *P* > 0.1 and *I*^2^ < 50%, the fixed-effects model was used to compute the combined ES. To the contrary, if *I*^2^ ≥ 50% and *P* < 0.1, suggesting high heterogeneity, choose a random-effects model. Subgroup analyses and multivariate meta-regression were performed to examine whether the impact and safety of ketamine and esketamine varied depending on moderating variables, including grades of shivering, administration methods (intravenous vs. epidural), anesthesia methods (spinal anesthesia, epidural anesthesia vs. combined spinal and epidural anesthesia), and dose. Furthermore, we conducted a leave-one-out analysis to assess the stability and sources of heterogeneity in the outcomes. Egger’s linear regression test was carried out by STATA 18.0 to evaluate the publication bias, with a *p* < 0.05 indicating a significant bias. Logistic regression with continuous dose modeling established dose-response relationships for the incidence of shivering. Pearson χ^2^ standard errors were utilized for statistical inference when the results suggested overdispersion.

## Results

### Literature search

**FIGURE 1:**
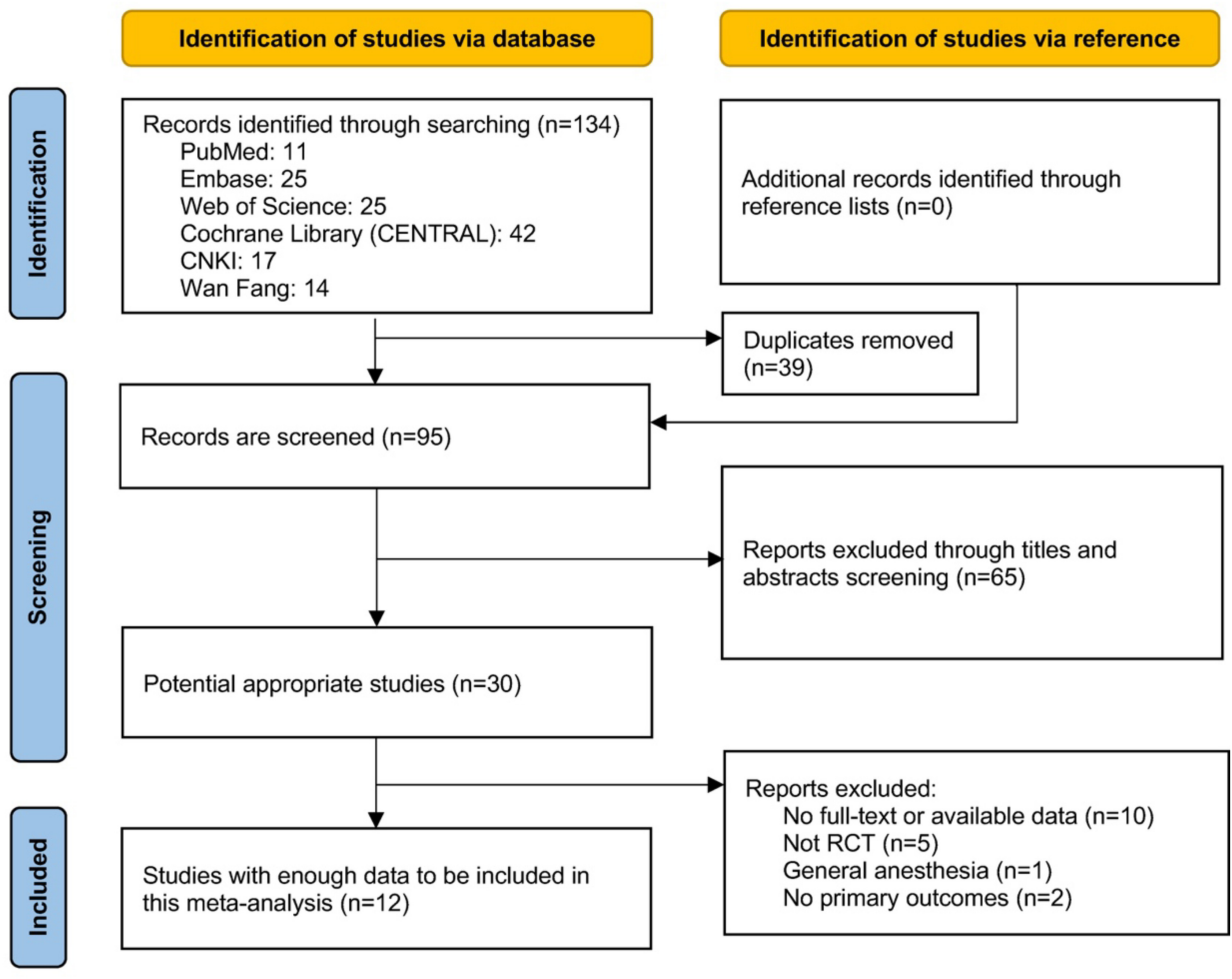
Flowchart showing the process of study selection for this meta-analysis.

A total of 134 records were retrieved, and 39 duplicate records were removed. 30 records were included after the title and abstract screening. Ultimately, 12 RCTs with 1683 patients were eligible by reading the full text and meeting the inclusion criteria (18–29), including three (23, 24, 28) on the use of esketamine and nine (18–22, 25–27, 29) on the use of ketamine. The flow diagram of the research selection process and details are shown in Figure 1.

### Characteristics of included studies

The main characteristics of the 12 studies in this meta-analysis are shown in Table 1. Participants in six studies (18–20, 22, 25, 29) underwent cesarean delivery under spinal anesthesia, five studies (21, 24, 26–28) under combined spinal-epidural anesthesia, and one study (23) under epidural anesthesia. The administrations in the included studies were different. Trial drugs were administered intravenously in 10 studies (18–20, 22, 23, 25–29) and were administered epidurally in the other two studies (21, 24). Regarding the measurement of shivering severity, seven studies (18–22, 26, 28) chose a rating scale ranging from 0 to 4, three studies (23, 24, 27) applied a 0–3 scale for evaluating the severity of shivering, and two studies (25, 29) did not assess the severity of shivering.

**TABLE 1:** Characteristics of included studies.

Study ID	Country	Participants		Anesthesia methods	Comparison groups	Definition of shivering	
		Baseline	*n*			Grades	Treatment
Kose (18) 2013	Turkey	ASA I-II 18–45	30/30/30	SA L_3–4_ or L_4–5_	IV: saline IV: 0.25 mg/kg K IV: 0.5 mg/kg K	0–4	Meperidine 25 mg iv
Xue X (26) 2013	China	ASA I-II 18–35	30/30/30	CSEA L_3–4_ or L_4–5_	IV: saline IV: 0.25 mg/kg K IV: 0.5 mg/kg K	0–4	Pethidine 25 mg iv
Mohtadi (19) 2016	Iran	ASA I 18–40	39/39/39	SA L_3–4_	IV: saline IV: 0.25 mg/kg K IV: 4 mg ondansetron	0–4	/
Jaafarpour (29) 2017	Iran	ASA I-II 20–35	23/23/23/23	SA L_3–4_	IV: saline IV: 0.25 mg/kg propofol IV: 0.25 mg/kg K IV: 25 mg K + propofol	NA	NA
Lema (20) 2017	Ethiopia	ASA I-II 18–39	41/41/41	SA L_3–4_ or L_4–5_	IV: saline IV: 0.2 mg/kg K IV: 0.5 mg/kg tramadol	0–4	Pethidine 25 mg iv
Wang Y (27) 2018	China	ASA I-II 20–45	39/40/38/36	CSEA L_3–4_ or L_4–5_	IV: saline IV: 0.05 mg/kg K IV: 0.1 mg/kg K IV: 0.25 mg/kg K	0–3	Tramadol 100 mg iv
Xue X (21) 2018	China	ASA I-II 22–41	30/30	CSEA L_3–4_	Epidural: saline Epidural: 0.5 mg/kg K	0–4	Meperidine 25 mg iv
Jouryabi (22) 2021	Iran	ASA II 18–40	127/127/127/127	SA L_3–4_ or L_4–5_	IV: saline IV: 0.2 mg/kg K IV: 0.5 mg/kg tramadol IV: 4 mg ondansetron	0–4	Pethidine 25 mg iv
Aboelsuod (25) 2023	Egypt	ASA II-III 21–40	63/63	SA L_4–5_	IV: saline IV: 0.3 mg/kg K	/	/
Jin MH (23) 2022	China	ASA I-II 20–40	27/28/26	EA L_2–3_	IV: saline IV: 0.25 mg/kg ES IV: 0.5 mg/kg ES	0–3	Tramadol 100 mg iv
Yang D (28) 2023	China	ASA II 20–40	28/28/29	CSEA L_3–4_	IV: saline IV: 0.15 mg/kg ES IV: 0.3 mg/kg ES	0–4	/
Zhang X (24) 2023	China	ASA II 18–40	79/79	CSEA L_2–3_	Epidural: saline Epidural: 0.25 mg/kg ES	0–3	/

SA, spinal anesthesia; EA, epidural anesthesia; CESA, combined spinal and epidural anesthesia; IV, intravenous injection; K, ketamine; ES, esketamine.

### Bias risk assessment

**FIGURE 2:**
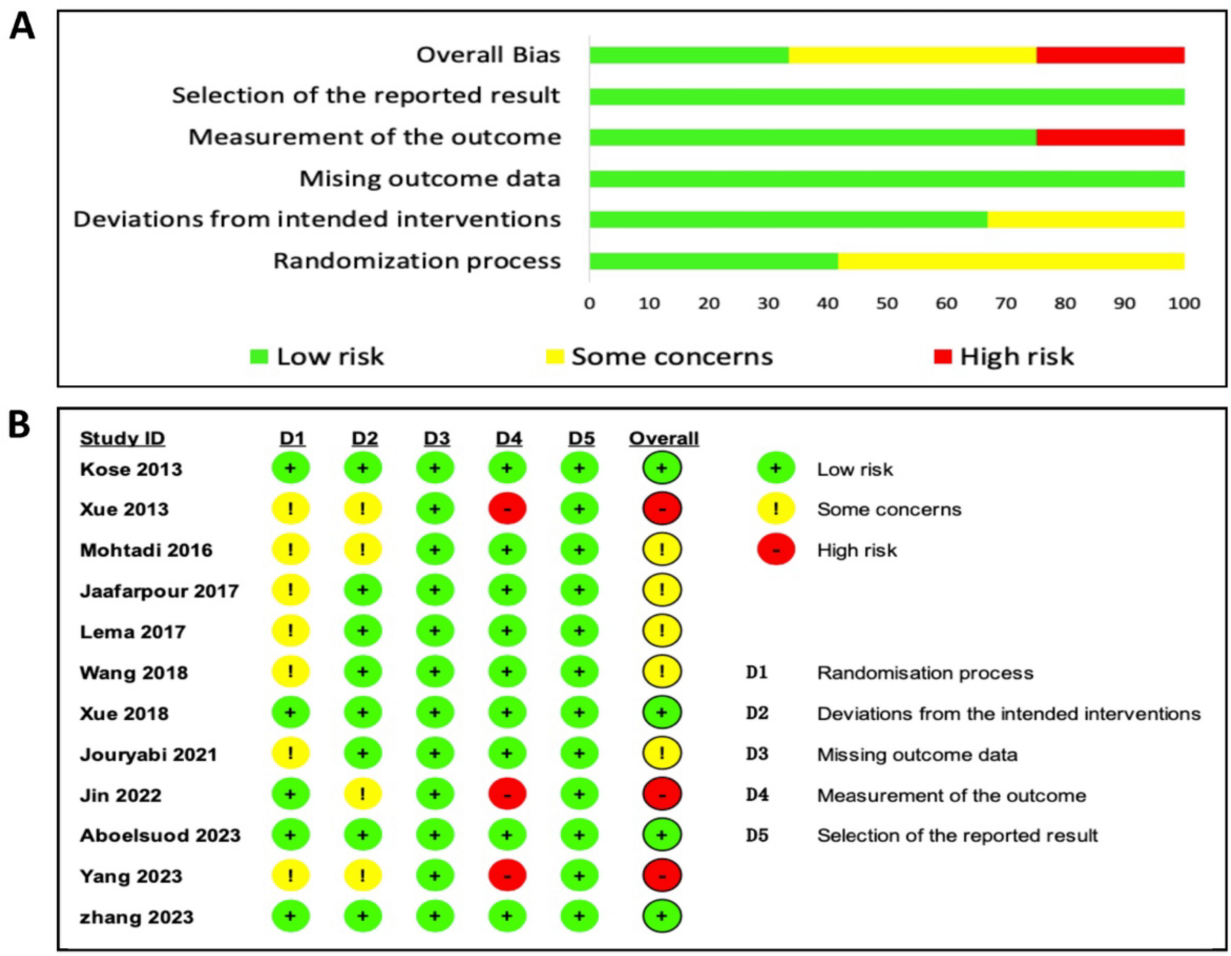
Risk of bias graph **(A)** and summary **(B)**.

We adopted the Cochrane Handbook to assess the risk of bias in RCTs, and the results of the quality assessment are presented in Figure 2. Seven studies (19, 20, 22, 26–29) were judged to be at “some concerns” because of a lack of description about the random sequence generation method and concealment. Four studies (19, 23, 26, 28) did not explain the specific implementation of blindness, and the outcome may have been affected by the lack and was assessed as “some concerns.” Three studies (23, 26, 28) did not specify exactly whether the outcome assessors were aware of the intervention received by participants and were rated as having “high risk.” To conclude, four studies (21, 24–26) were judged to be at low risk of overall bias; five studies (19, 20, 22, 27, 29) were found to have some concerns regarding the overall bias; and the remaining three studies (23, 26, 28) were considered to have a high risk due to the bias in the measurements of the outcomes and the implementation of blindness.

### The effect of ketamine and esketamine vs. saline on the prevention of shivering

**FIGURE 3:**
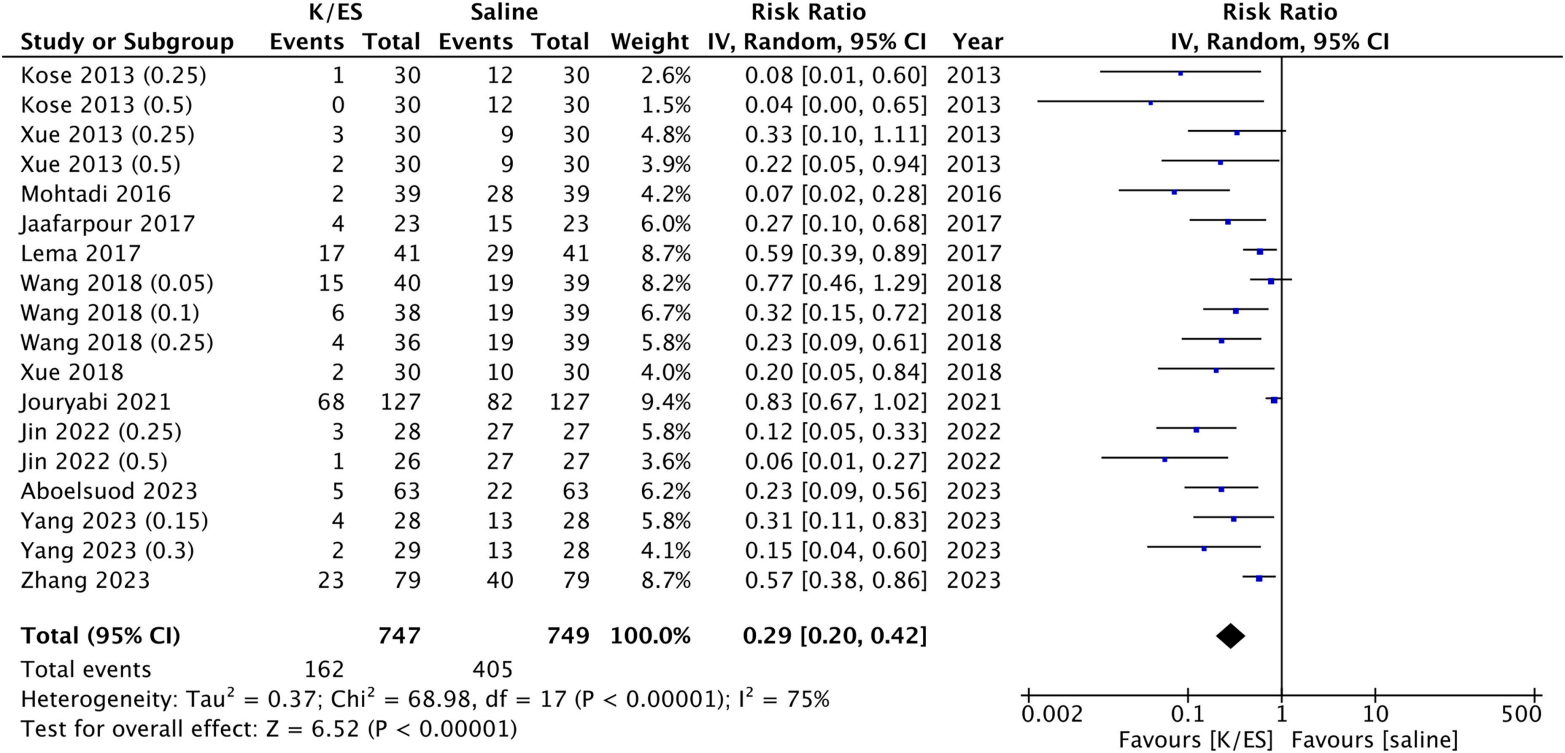
Forest plots of the incidence of postanesthetic shivering.

12 RCTs reported the incidence of postanesthetic shivering in parturients with ketamine (18–22, 25–27, 29) and esketamine (23, 24, 26) administration. The random-effects model was utilized because the value of *I*^2^ > 50%. Ketamine and esketamine have been found to significantly decrease the incidence of postanesthetic shivering (RR: 0.29; 95% CI: 0.20–0.42; *P* < 0.00001; *I*^2^ = 75%, *P* < 0.00001) compared to saline (Figure 3).

**FIGURE 4:**
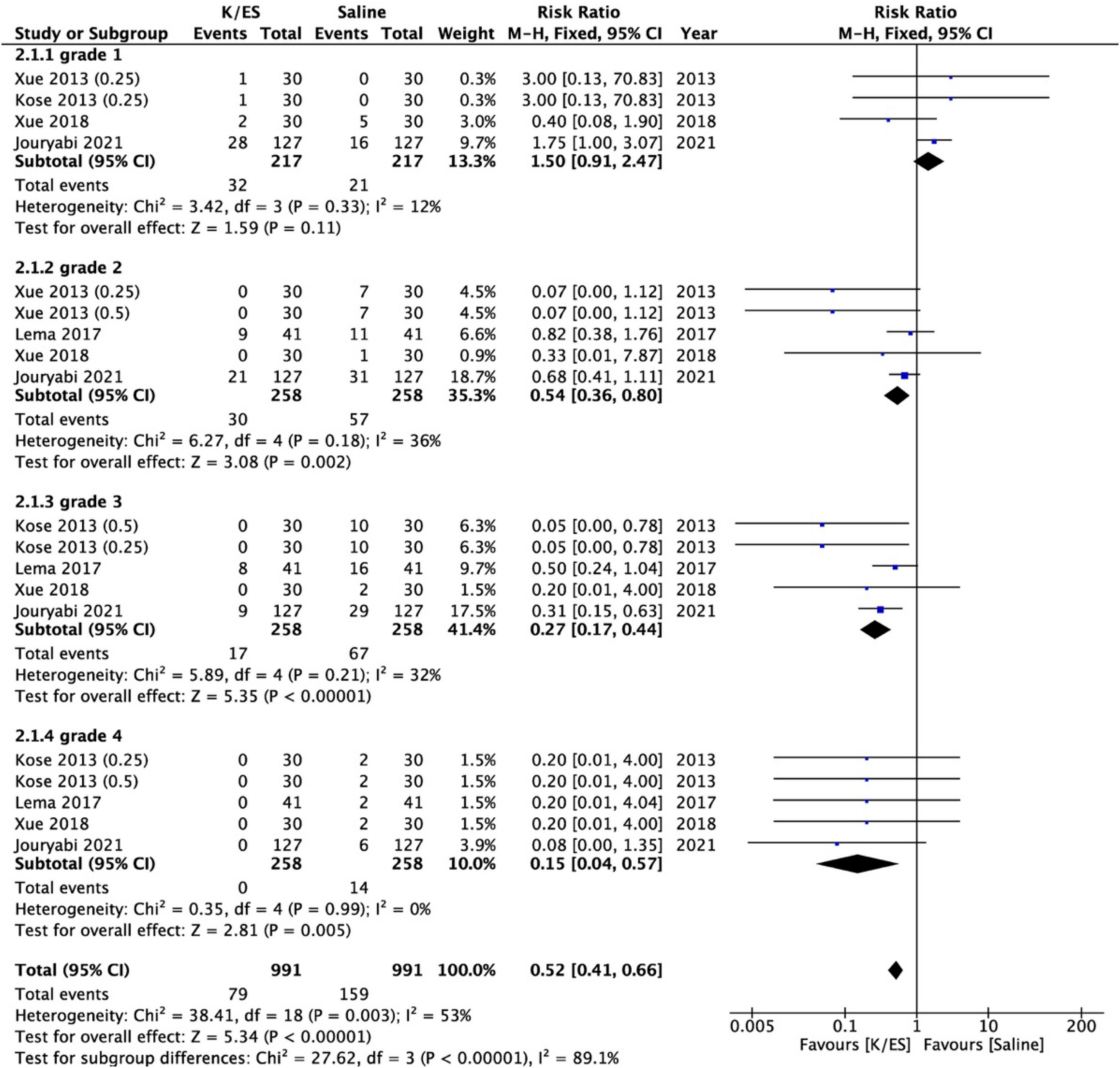
Subgroup analysis of the grades of the postanesthetic shivering.

Subgroup analyses were performed to explore the potential reasons regarding the prominent heterogeneity. A total of five studies (18, 20–22, 26) observed the severity of shivering, which can be divided into five grades (0–4). The results showed that the administration of ketamine significantly reduced the postanesthetic shivering for grade 2 (RR: 0.54; 95% CI: 0.36–0.80; *P* = 0.002; *I*^2^ = 36%, *P* = 0.18), grade 3 (RR: 0.27; 95% CI: 0.17–0.44; *P* < 0.00001; *I*^2^ = 32%, *P* = 0.21), and grade 4 (RR: 0.15; 95% CI: 0.04–0.57; *P* = 0.005; *I*^2^ = 0%, *P* = 0.99). However, there was no significant difference for grade 1 (RR: 1.50; 95% CI: 0.91–2.47; *P* = 0.11; *I*^2^ = 12%, *P* = 0.33) (Figure 4). A significant subgroup difference was observed between different shivering grades (*P* < 0.00001), and the internal heterogeneity of each subgroup was not significant, which may explain part of the high heterogeneity of the primary outcomes.

Based on the different administrations, only intravenous injection (IV) exhibited a significant reduction in the incidence of postanesthetic shivering (RR: 0.28; 95% CI: 0.18–0.42; *P* < 0.00001; *I*^2^ = 77%, *P* < 0.00001) (Table 2 and Supplementary Figure 1). Regardless of the anesthesia methods (spinal anesthesia, epidural anesthesia vs. combined spinal and epidural anesthesia), the incidence of postanesthetic shivering was significantly reduced (Supplementary Figure 2).

**TABLE 2:** Subgroup analyses of the incidence of postanesthetic shivering.

Variables	*n*	RR	95% CI	*P*	*I* ^2^	Heterogeneity test (*P*)
1. Anesthetic methods						
SA	7	0.29	[0.15, 0.55]	0.0002	81%	< 0.0001
EA	2	0.10	[0.04, 0.23]	< 0.00001	0%	0.41
CSEA	9	0.38	[0.26, 0.56]	< 0.00001	40%	0.10
2. Administrations						
IV	16	0.28	[0.18, 0.42]	< 0.00001	77%	<0.00001
Epidural	2	0.43	[0.17, 1.08]	0.07	48%	0.16

RR, risk ratio; SA, spinal anesthesia; EA, epidural anesthesia; CESA, combined spinal and epidural anesthesia; IV, intravenous injection.

### The effect of ketamine and esketamine vs. saline on the incidence of adverse events

**FIGURE 5:**
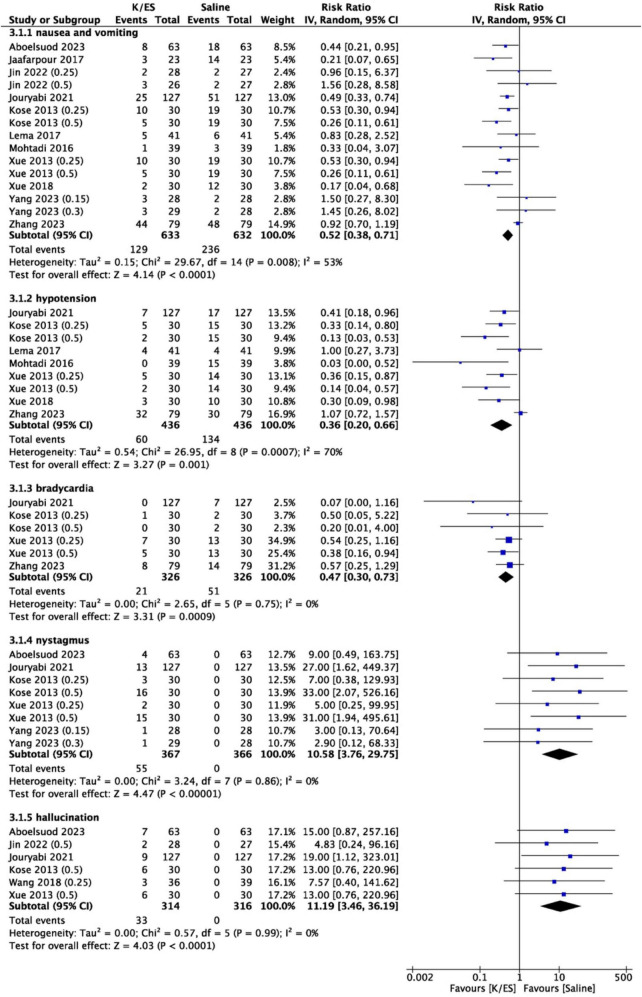
Forest plot of the risk of adverse events.

All 12 RCTs reported the adverse events, and we analyzed the five common adverse events that included nausea and vomiting, hypotension, bradycardia, nystagmus, and hallucination. Ketamine and esketamine reduced the incidence of nausea and vomiting (RR: 0.52; 95% CI: 0.38–0.71; *P* < 0.0001; *I*^2^ = 53%, *P* = 0.008), hypotension (RR: 0.36; 95% CI: 0.20–0.66; *P* = 0.001; *I*^2^ = 70%, *P* = 0.0007), and bradycardia (RR: 0.47; 95% CI: 0.30–0.73; *P* = 0.0009; *I*^2^ = 0%, *P* = 0.75) compared with the placebo. However, the incidence of nystagmus (RR: 10.58; 95% CI: 3.76–29.75; *P* < 0.00001; *I*^2^ = 0%, *P* = 0.86) and hallucinations (RR: 11.19; 95% CI: 3.46–36.19; *P* < 0.0001; *I*^2^ = 0%, *P* = 0.99) was significantly higher in the patients who received ketamine/esketamine than in those who received placebo (Figure 5).

### Sensitivity analysis

To explore the sources of heterogeneity, leave-one-out sensitivity analysis was conducted on the primary outcomes of the incidence of shivering and subgroups of the intravenous administration and spinal anesthesia. Furthermore, regarding the high heterogeneity, secondary outcomes of the incidence of nausea and vomiting and hypotension were also performed with a sensitivity analysis. The results were stable and reliable (Supplementary Figures 3–7).

### Multivariate meta-regression

A multivariate meta-regression was performed to explore potential sources of heterogeneity of the outcome “incidence of shivering,” where we regarded dose, different drugs, administrations, and anesthesia methods as covariates. The joint test for all covariates was not statistically significant (*P* = 0.091), although the model explained 52% of the between-study variance (adjusted *R*^2^ = 0.52). Among the covariates, only dose was significantly associated with the effect size (*P* = 0.028), indicating that dose may be an important moderator (Supplementary Figure 8).

### Logistic regression

**FIGURE 6:**
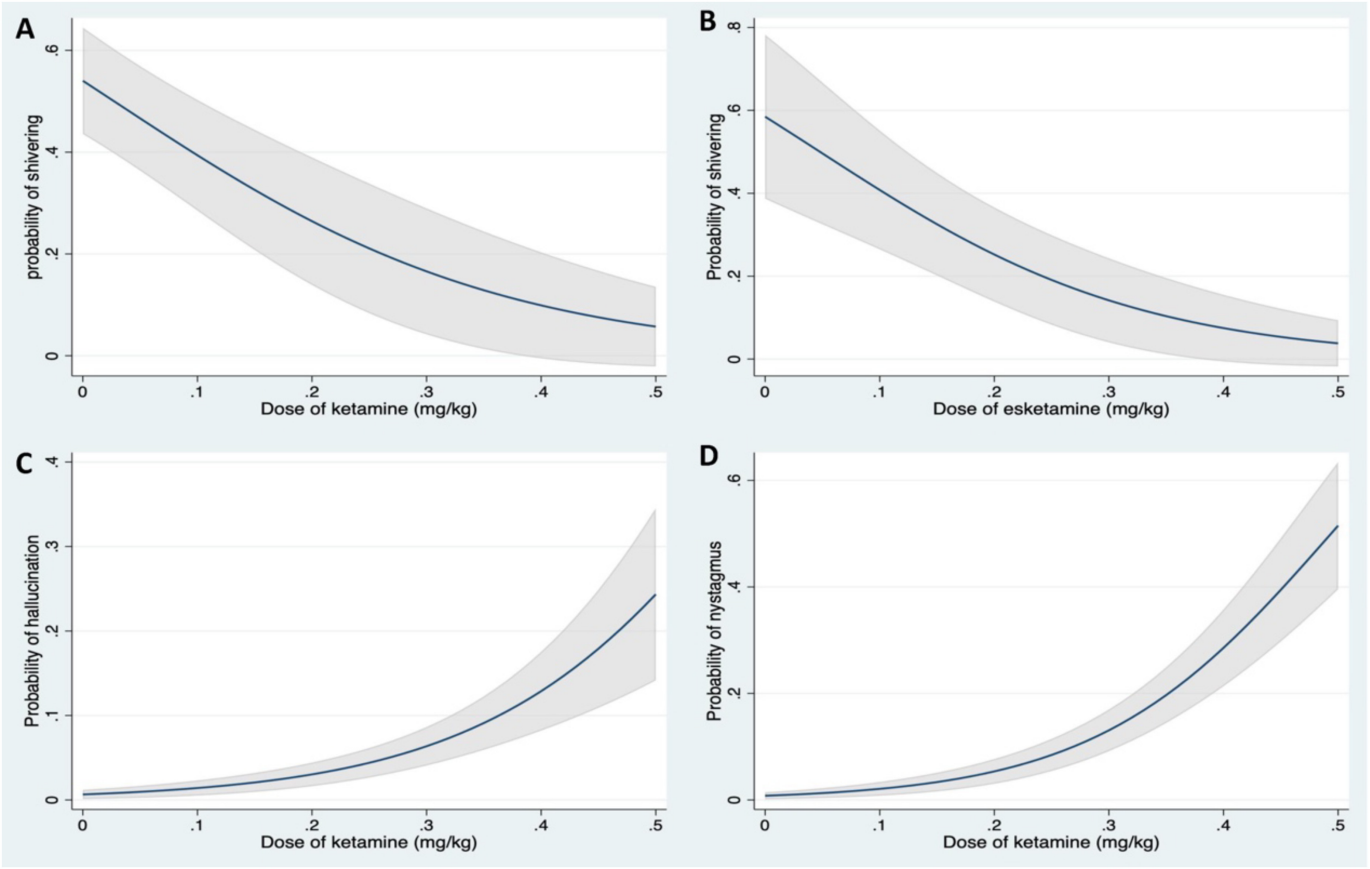
Dose-response curves for the incidence of shivering and adverse events. **(A)** shivering vs. ketamine dose; **(B)** shivering vs. esketamine dose; **(C)** hallucination and **(D)** nystagmus vs. the ketamine dose.

In view of the meta-regression results, we further explore the dose-response relationship between doses and the incidence of shivering. Logistic regression with generalized linear models was used to evaluate the dose-response relationship and provided a 95% confidence interval. Figures 6A,B showed the relationship between the probability of shivering and the dose of ketamine and esketamine, respectively. Each 0.1 mg/kg increase in ketamine was associated with a 45% reduction in the odds of shivering (OR: 0.55; 95% CI: 0.42–0.73; *P* < 0.001). The odds of shivering declined by 51% per 0.1 mg/kg increment in esketamine dose (RR: 0.49; 95% CI: 0.31–0.78; *P* = 0.003). In addition, it is noted that the incidence of hallucinations (Figure 6C) and nystagmus (Figure 6D) rose significantly with the increase in the ketamine dose (*P* < 0.001).

### Publication bias

We used Egger’s test and the funnel plots to assess publication bias. The funnel plots were found to be asymmetrical (*P* < 0.05) in the incidence of postanesthetic shivering. As shown in Supplementary Figure 9, the merging effect is still stable after the trim and fill, suggesting that the conclusion is less sensitive to potential publication bias.

### Quality of evidence

We used the GRADE-pro guideline development tool to assess the quality of evidence for the primary and secondary outcomes of the included studies in this meta-analysis. Due to the risk of bias, imprecision, and high heterogeneity, the quality of the evidence was mostly low and moderate. The summary of findings and quality of evidence is shown in Supplementary Table 2.

## Discussion

Our meta-analysis indicated that ketamine and esketamine can effectively prevent postanesthetic shivering during cesarean delivery, particularly for grade 2–4 shivering. There is a significant dose-response relationship between shivering incidence and the dose. Ketamine and esketamine had an advantage in reducing the incidence of nausea and vomiting, hypotension, and bradycardia compared with the placebo. In contrast, they were associated with some short-term adverse events of hallucination and nystagmus.

It was reported that approximately half of patients experienced postanesthetic shivering during cesarean delivery under intrathecal anesthesia (1, 30, 31). Although shivering is an automatic protective reflex that increases the generation of body heat through muscle contraction, it may increase oxygen consumption, influence the contraction of the uterus, and interfere with vital signal monitoring (20, 30). As NMDA receptor antagonists, ketamine and esketamine are likely to modulate thermoregulation at multiple levels. Ketamine inhibits signal transduction in the hypothalamus and related thermoregulatory centers by noncompetitively blocking NMDA receptors (32). Meanwhile, the blocking of NMDA receptors can regulate the activity of noradrenergic and serotonergic neurons in the locus coeruleus, inhibit the reuptake of norepinephrine after the ganglion, and then reduce the heat loss from the core to the periphery of the body to play an anti-shivering effect (32–34). Partial stimulation of ketamine on opioid μ-receptors may increase the threshold of tolerance to cold (35, 36). In addition, the sympathetic excitation of esketamine and ketamine can contract blood vessels and limit heat redistribution, thus reducing the occurrence of shivering (37, 38).

In this updated analysis, we expanded the scope of the literature search to include relevant studies that specifically focus on the incidence and severity of postanesthetic shivering during cesarean delivery. Additionally, we evaluated the anti-shivering effect of esketamine, which was not covered in the previous meta-analysis. Our research indicated that ketamine and esketamine have good efficacy in preventing postanesthetic shivering during cesarean delivery, which is basically consistent with the results of a recent network analysis (OR: 0.39; *P* = 0.01; *I*^2^ = 63%; *n* = 600) (17). In a meta-analysis of 16 studies, Zhou et al. (15) reported that ketamine significantly reduced the incidence of postanesthetic shivering, yet this protective effect was not observed in the cesarean delivery subgroup (OR: 0.12; *P* = 0.19; *I*^2^ = 92%; *n* = 120). It should be noted, however, that their conclusions could be compromised by a relatively small sample size and high heterogeneity. Subgroup analyses based on shivering grade supported the primary results, showing that ketamine particularly lowered the incidence of postanesthetic shivering for grades 2–4. We conducted a multivariable meta-regression analysis with several factors entered as covariates (e.g., different doses, administrations, anesthesia methods, and drugs). Our results revealed that dose was the only covariate significantly correlated with the effect among the covariates, pointing to its potential role as a key moderator. Based on this finding, we used logistic regression to explore the dose-response relationship between dose and the anti-shivering effect. A significant dose-response correlation was observed between the preventive efficacy of ketamine and esketamine and dose.

Furthermore, we evaluated the adverse events after the administration of ketamine and esketamine. Hypotension during cesarean delivery could lead to intermittent hypoperfusion of the vestibular system, causing nausea and vomiting (39, 40). Ketamine and esketamine showed an advantage in reducing the incidence of nausea and vomiting, hypotension, and bradycardia. However, they significantly increased the risk of hallucination and nystagmus, which aligns with previous studies (15, 17, 41). Wang et al. (17) reported that the ED_50_ and ED_95_ of ketamine when women experienced neuropsychiatric adverse effects are 0.273 and 0.761 mg/kg, respectively. Given the dose-dependent relationship of these neuropsychiatric adverse events, administering ketamine and esketamine at subanesthetic doses to obstetric patients may be a safer option. Recent meta-analyses have indicated that the intraoperative administration of a sub-anesthetic esketamine could reduce the incidence of severe postpartum depression by nearly 50% (41, 42). In addition, as an adjunctive analgesic, esketamine also effectively contributes to perioperative pain management and postpartum recovery (43). Therefore, while the short-term complications are undeniably distressing, a thorough risk-benefit assessment of ketamine and esketamine in obstetrics should incorporate their thermoregulatory properties, sympathomimetic effects, analgesic ability, and long-term antidepressant potential. Further high-quality multicenter studies are warranted to explore the optimal dosage of ketamine and esketamine and confirm the clinical value in obstetric anesthesia.

There are some limitations in this meta-analysis. First, the included studies had small sample sizes and high heterogeneity, potentially restricting the generalizability of our conclusions. Second, flaws in the randomization and blinding procedures, as well as in the outcome measurements, may have compromised the accuracy of our results. Third, although the quality of the evidence for our outcomes was mostly low, our results have clearly demonstrated that ketamine and esketamine were effective in preventing postanesthetic shivering. Fourth, while our meta-analysis relied on a generalized linear model for the dose-response relationship, exploring potential nonlinearity would help refine the treatment window for ketamine and esketamine in preventing shivering. Hence, these limitations highlight the need for further high-quality multicenter studies to verify and refine our findings.

## Conclusion

Overall, ketamine and esketamine are generally effective in preventing postanesthetic shivering among patients undergoing cesarean delivery, particularly for grades 2–4 of shivering. For parturients who experience hypotension and bradycardia after anesthesia, the sympathomimetic effects of these agents may even outweigh the risk of short-term neuropsychiatric adverse events. To ensure the comfort and safety of parturients, further studies are warranted to determine the optimal dose and explore other anti-shivering strategies.

## Data Availability

The original contributions presented in this study are included in this article/Supplementary material, further inquiries can be directed to the corresponding author.
