# Supplementary material for: Efficacy and safety of ketamine and esketamine for the prevention of postanesthetic shivering in cesarean delivery: a systematic review and meta-analysis
Source: Front Med (Lausanne). 2026 Jun 26;13:1845684. doi: 10.3389/fmed.2026.1845684 (PMC13350040; doi:10.3389/fmed.2026.1845684)

**Supplemental materials**

| **Table S1.** Search strategies | |
| --- | --- |
| PubMed  (11) | ("shiverers"[All Fields] OR "shivering"[MeSH Terms] OR "shivering"[All Fields] OR "shiver"[All Fields] OR "shivered"[All Fields] OR "shiverer"[All Fields] OR "shiverings"[All Fields] OR "shivers"[All Fields]) AND ("ketamin"[All Fields] OR "ketamine"[Supplementary Concept] OR "ketamine"[All Fields] OR "esketamine"[Supplementary Concept] OR "esketamine"[All Fields] OR "esketamine"[Supplementary Concept] OR "esketamine"[All Fields] OR "ketamine"[MeSH Terms] OR "ketamine s"[All Fields] OR "ketamines"[All Fields]) AND ("cesarean section"[MeSH Terms] OR ("cesarean"[All Fields] AND "section"[All Fields]) OR "cesarean section"[All Fields] OR ("cesarean"[All Fields] AND "delivery"[All Fields]) OR "cesarean delivery"[All Fields]) |
| Web of Science  (25) | ((TS=(ketamine OR Esketamine OR L-Ketamine OR S-Ketamine OR Spravato)) AND TS=(shivering)) AND TS=(cesarean section) |
| Embase  (25) | (ketamine:ti,ab,kw OR 'esketamine'/exp OR 'l ketamine':ti,ab,kw OR 's ketamine':ti,ab,kw OR spravato:ti,ab,kw) AND (shivering:ti,ab,kw) AND ('cesarean section':ti,ab,kw) |
| Cochrane library  (42) | **((ketamine):ab,kw,ti OR** (S-ketamine):ab,kw,ti OR (esketamine):ab,kw,ti) AND (shivering):**ab,kw,ti)** AND (cesarean section):ab,kw,ti |
| CNKI  (17) | （主题：氯胺酮）OR（主题：艾司氯胺酮）AND（主题：寒战）AND（主题：剖宫产） |
| Wan Fang  (14) | （题名或关键词:(氯胺酮) or 题名或关键词:(艾司氯胺酮)） and 题名或关键词:(寒战) and 题名或关键词:(剖宫产) |

| **Table S2**  GRADE evidence profile and summary of findings | | | | | | |
| --- | --- | --- | --- | --- | --- | --- |
| ***n*** | Study  design | **No. of patients** | | **Effect** | | **Certainty** |
|  |  | K/ES | placebo | Relative (95% CI) | Absolute (95% CI) |  |
| **Incidence of postanesthetic shivering-ketamine** | | | | | | |
| 13 | RCT | 129/557  (23.3%) | 285/560  (50.9%) | **RR 0.33**  (0.21, 0.50) | 341 fewer per 1000 (402 to 254) | **⊕⊕OO**  Low |
| **Incidence of postanesthetic shivering-esketamine** | | | | | | |
| 5 | RCT | 33/190  (17.4%) | 390/726  (53.7%) | **RR** **0.28**  (0.18, 0.43) | 387 fewer per 1000 (440 to 306) | **⊕⊕OO**  Low |
| **Postanesthetic shivering for grade 1** | | | | | | |
| 4 | RCT | 32/217  (14.7%) | 21/217  (9.7%) | **RR** **1.50**  (0.91, 2.47) | 48 more per 1000  (9 to 142) | **⊕OOO**  Very Low |
| **Postanesthetic shivering for grade 2** | | | | | | |
| 5 | RCT | 30/258  (11.6%) | 57/258  (22.1%) | **RR 0.54**  (0.36, 0.80) | 102 fewer per 1000  (141 to 44) | **⊕⊕OO**  Low |
| **Postanesthetic shivering for grade 3** | | | | | | |
| 5 | RCT | 17/258  (6.6%) | 67/258  (26.0%) | **RR 0.27**  (0.17, 0.44) | 190 fewer per 1000 (216 to 145) | **⊕⊕⊕O**  Moderate |
| **Postanesthetic shivering for grade 4** | | | | | | |
| 5 | RCT | 0/258  (0.0%) | 14/258  (5.4%) | **RR** **0.15**  (0.04, 0.57) | 46 fewer per 1000  (52 to 23) | **⊕⊕OO**  Low |
| **Nausea and vomiting** | | | | | | |
| 15 | RCT | 129/633  (20.4%) | 236/632  (37.3%) | **RR** **0.52**  (0.38, 0.71) | 179 fewer per 1000 (232 to 108) | **⊕⊕OO**  Low |
| **Hypotension** | | | | | | |
| 9 | RCT | 60/436  (13.8%) | 134/436  (30.7%) | **RR** **0.35**  (0.18, 0.68) | 200 fewer per 1000 (252 to 98) | **⊕OOO**  Very low |
| **Bradycardia** | | | | | | |
| 6 | RCT | 21/326  (6.4%) | 51/326  (15.6%) | **RR** **0.47**  (0.30, 0.73) | 83 fewer per 1000  (110 to 42) | **⊕⊕⊕O**  Moderate |
| **Nystagmus** | | | | | | |
| 8 | RCT | 55/367  (15.0%) | 0/366  (0.0%) | **RR** **10.58**  (3.76, 29.75) | 0 fewer per 1000  (0 to 0) | **⊕⊕⊕O**  Moderate |
| **Hallucination** | | | | | | |
| 5 | RCT | 33/314  (10.5%) | 0/316  (0.0%) | **RR** **11.19**  (3.46, 36.19) | 0 fewer per 1000  (0 to 0) | **⊕⊕⊕O**  Moderate |
| ***n*:** number of studies; **RCT:** randomized controlled trial; **K:** ketamine; **ES:** esketamine; **RR:** risk ratio; **CI:** confidence interval. | | | | | | |

**Figure S1.** Subgroup analysis for the incidence of postanesthetic shivering according to the different administrations. **IV:** intravenous injection.

**
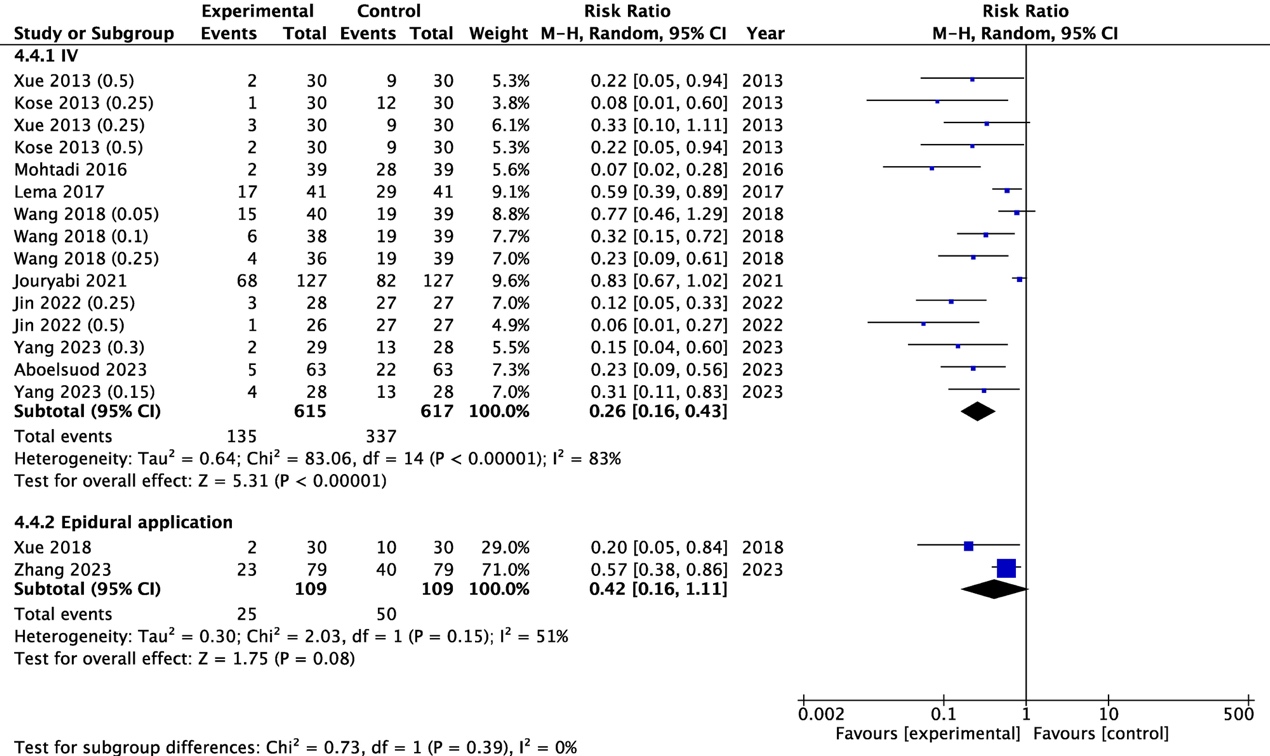
**

**Figure S2.** Subgroup analysis for the incidence of postanesthetic shivering according to the different anesthesia methods. **SA:** spinal anesthesia; **EA:** epidural anesthesia; **CSEA:** combined spinal-epidural anesthesia.

**
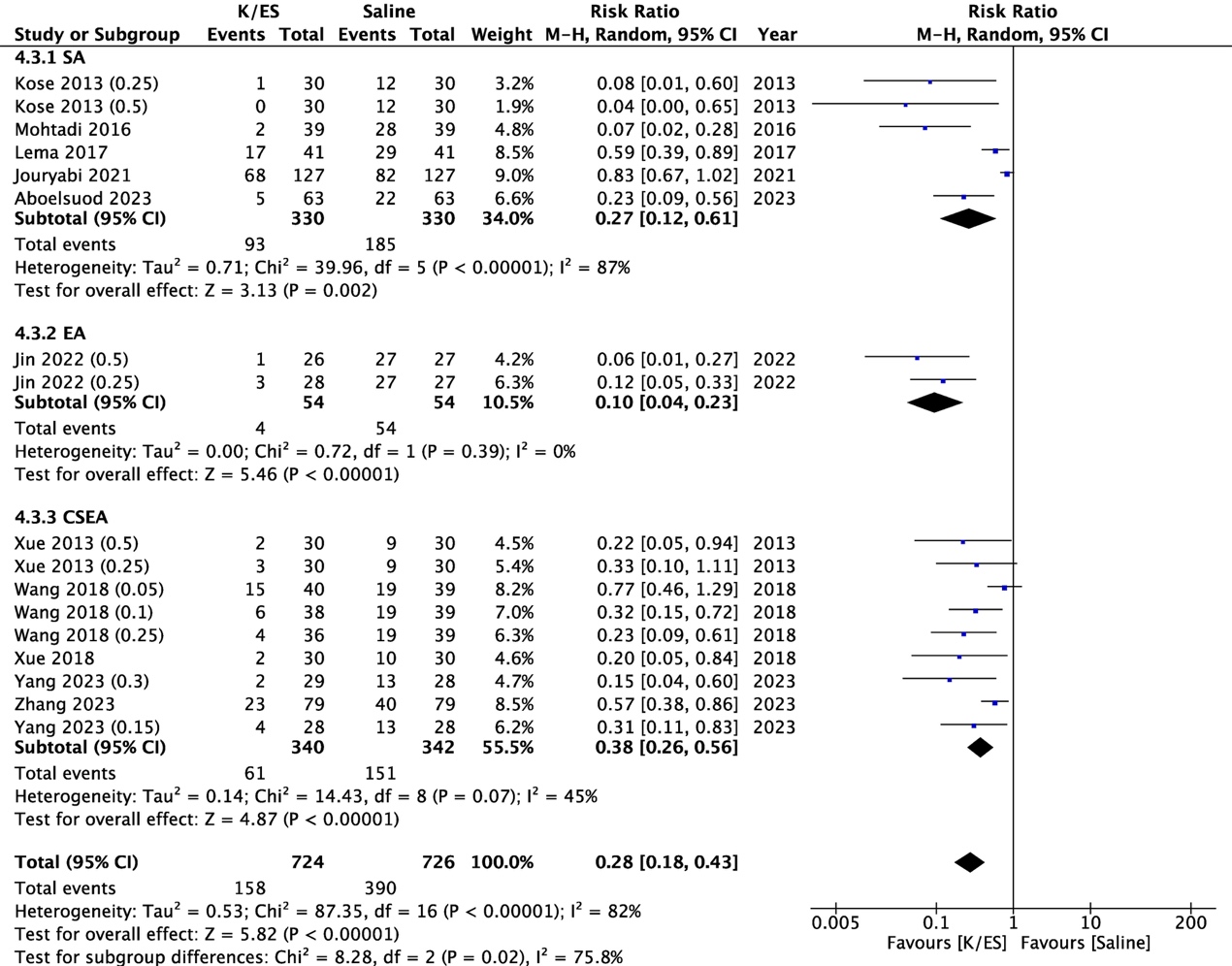
**

**Figure S3.** Leave-one-out analysis results for the incidence of postanesthetic shivering.


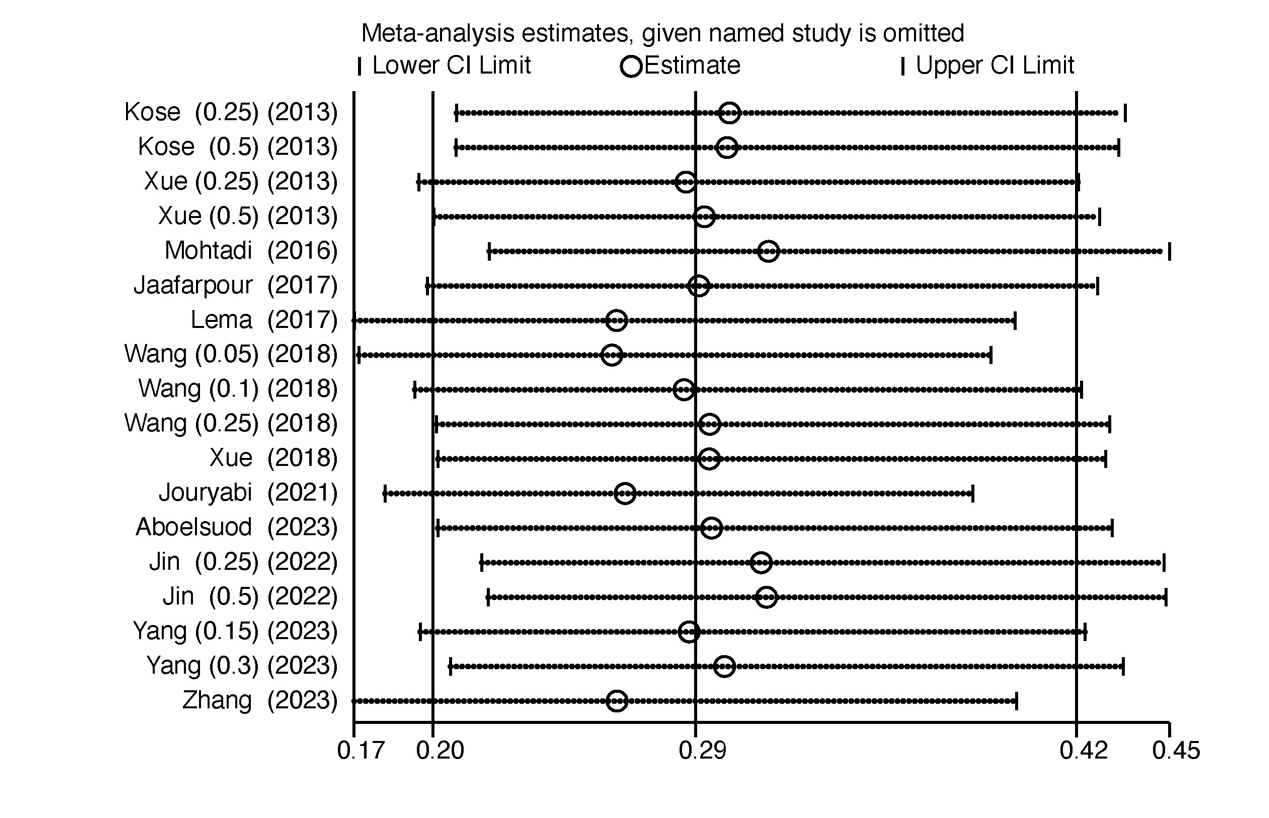


**Figure S4.** Leave-one-out analysis results for the subgroup analyses of the intravenous administration.


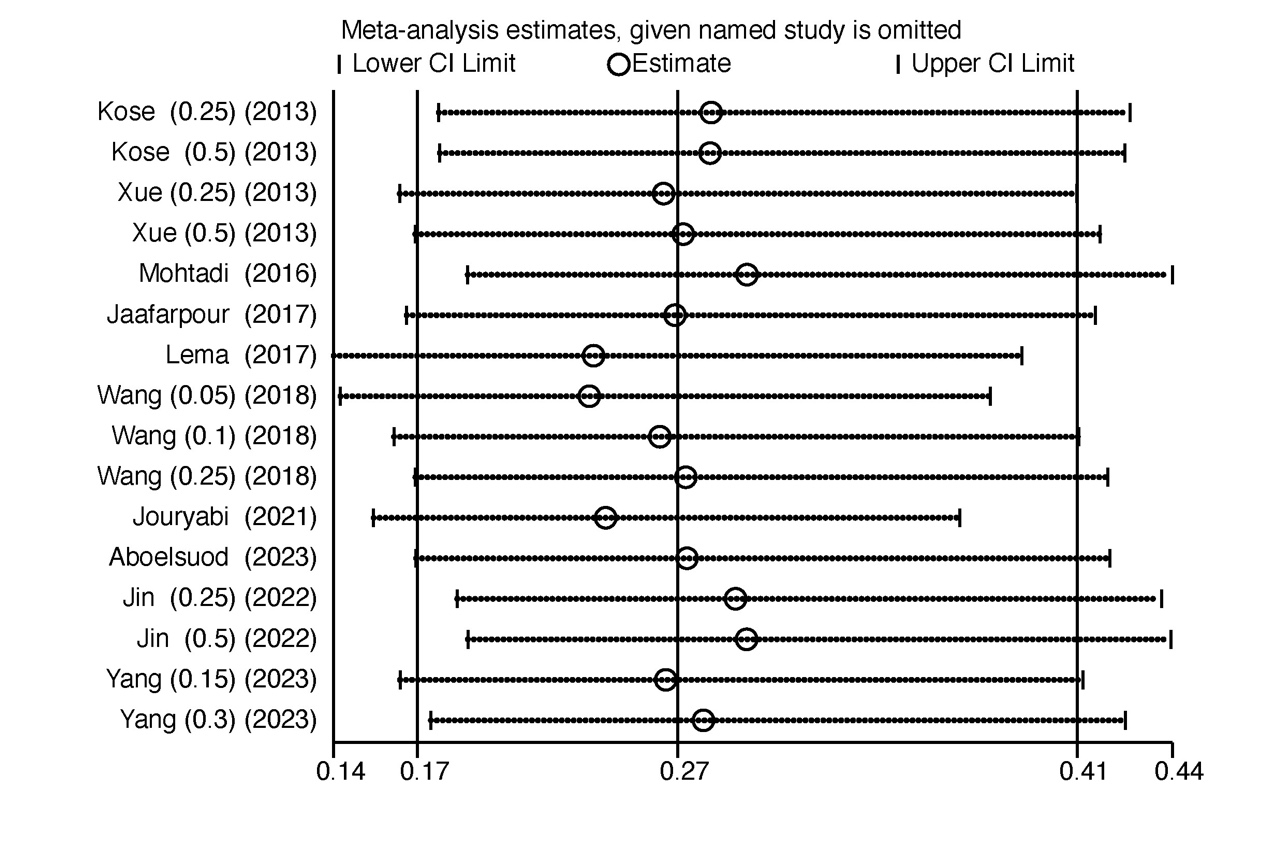


**Figure S5.** Leave-one-out analysis results for the subgroup analyses of the spinal anesthesia.

**
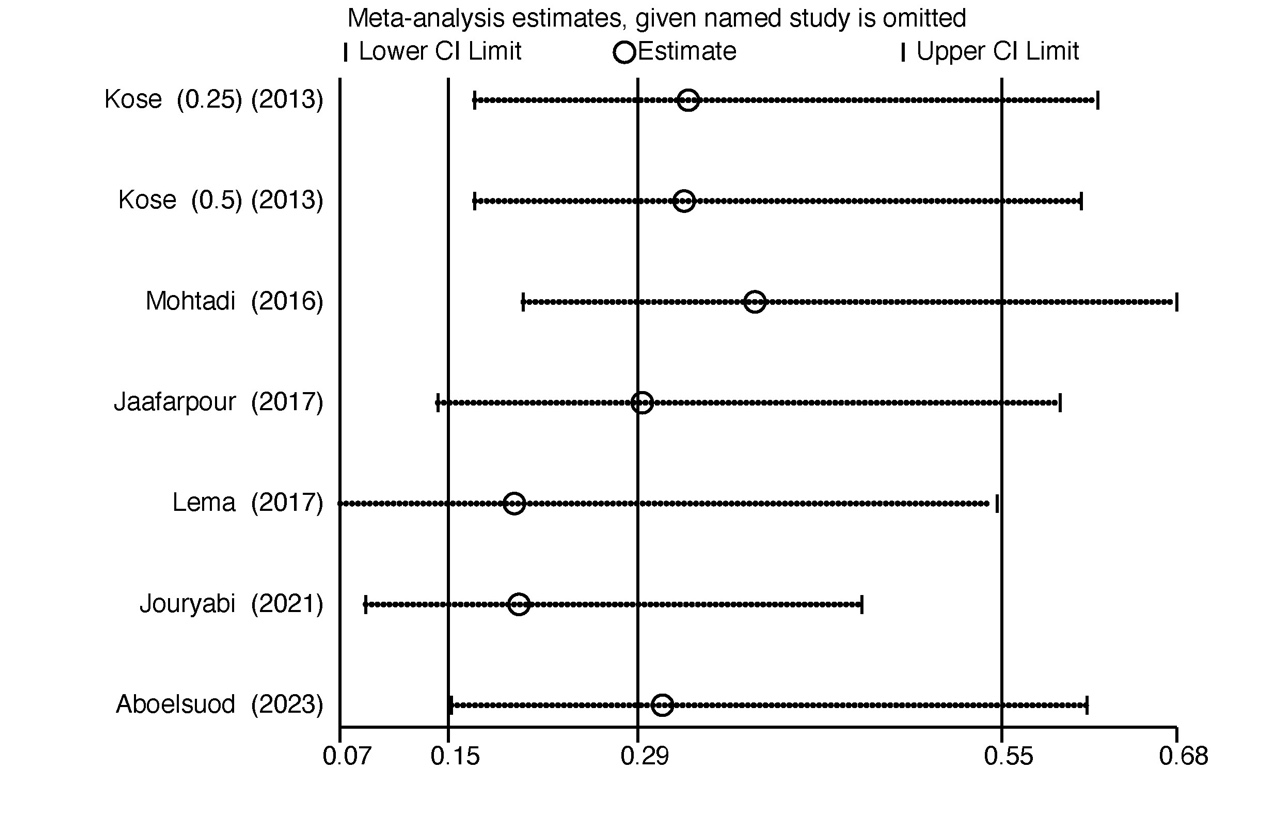
**

**Figure S6.** Leave-one-out analysis results for the incidence of nausea and vomiting.

**
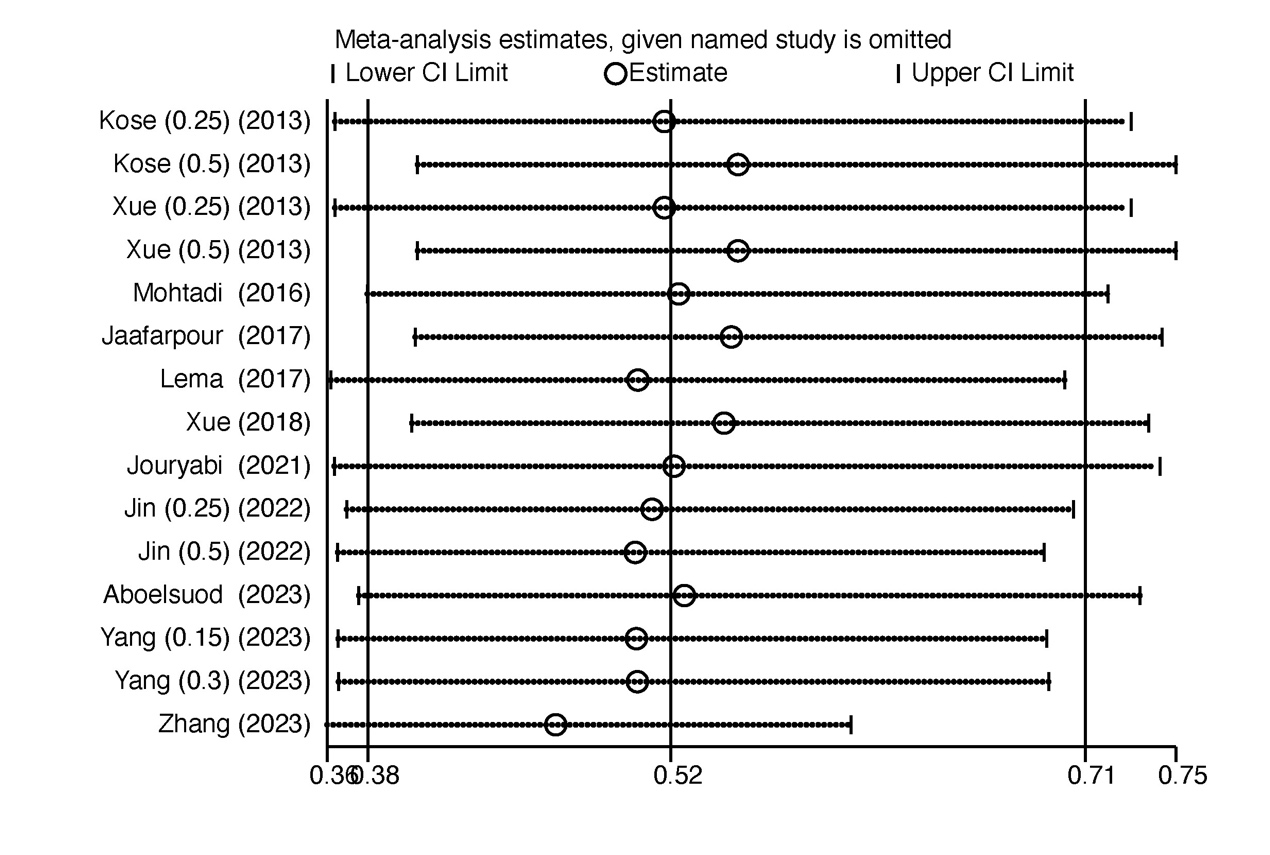
**

**Figure S7.** Leave-one-out analysis results for the incidence of hypotension.

**
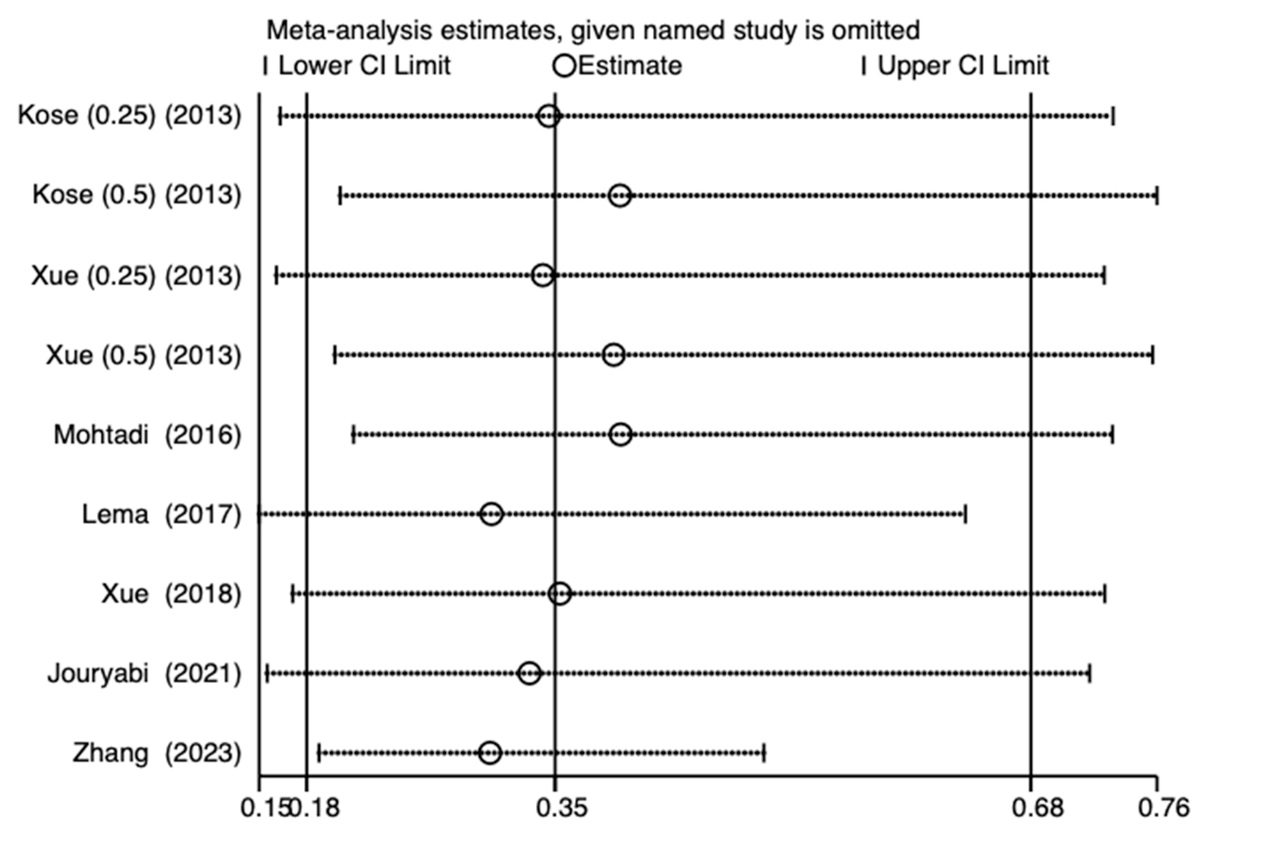
**

**Figure S8.** Results of the multivariate meta-regression.

**
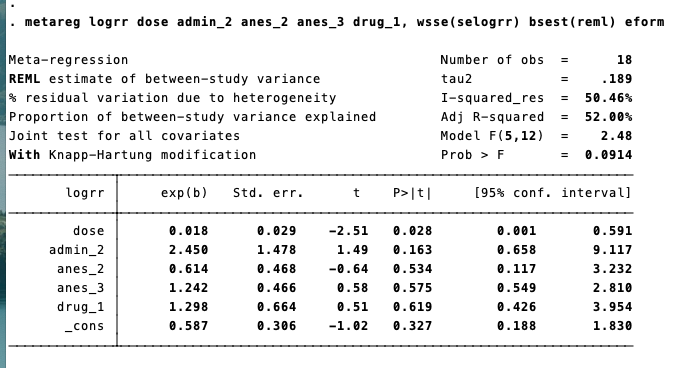
**

**Figure S9.** The trim-and-fill analysis results **(A)** and funnel plots **(B)**.


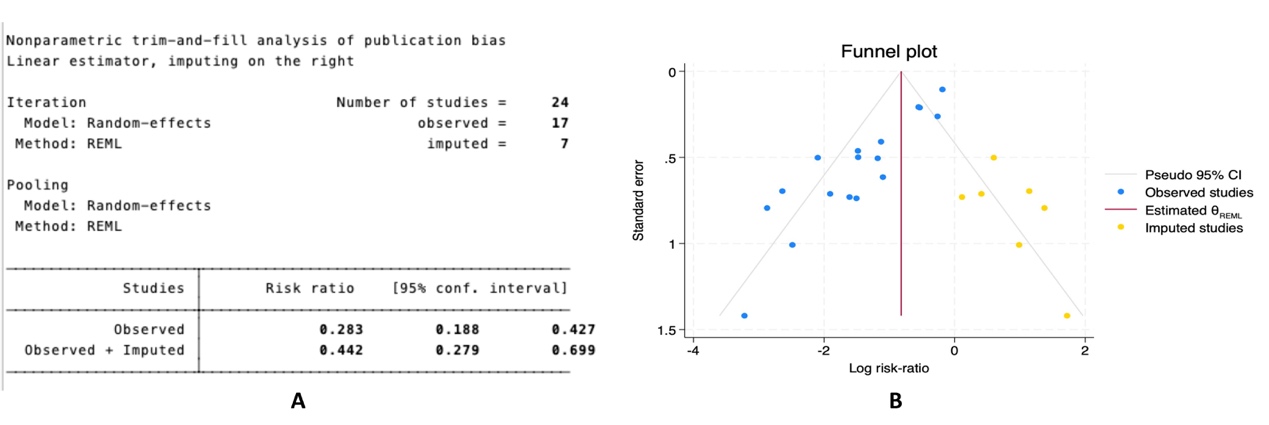

Supplement: Supplementary file 1 [file Supplementary_file_1.docx]
